# Early Tracheostomy in Morbidly Obese COVID-19 Patients: A Case Series and Discussion of Institutional Practices

**DOI:** 10.7759/cureus.14345

**Published:** 2021-04-07

**Authors:** Mathew P Caputo, Steven Aziz, Matthew Mifsud

**Affiliations:** 1 Otolaryngology-Head and Neck Surgery, University of South Florida Morsani College of Medicine, Tampa, USA

**Keywords:** endotracheal intubation, tracheostomy, covid-19, morbid obesity, sars-cov-2

## Abstract

Tracheostomies are often utilized in critically ill patients on prolonged mechanical ventilation, to enhance respiratory function and facilitate ventilator weaning. Many coronavirus disease 2019 (COVID-19) patients develop serious respiratory illness requiring ventilator management. In the early phase of this pandemic, the risk of disease spread lead to the development of conservative guidelines which advocated delaying tracheostomy at least two to three weeks from intubation and, preferably, with negative COVID-19 testing. The morbidly obese patient population, however, presents a unique scenario in which early tracheostomy may be beneficial. In this article, we discuss our institution’s current practices along with clinical outcomes with reference to intensive care literature and propose that early tracheotomy in COVID-19 patients should be considered on a case by case basis.

## Introduction

As coronavirus disease 2019 (COVID-19) has rapidly spread across the globe, quickly achieving pandemic status, healthcare institutions have been forced to rapidly alter their practices in order to limit potential spread among health care workers. Although about 80% of patients with COVID-19 have a relatively mild/self-limiting illness, roughly five percent will become critically ill, developing an illness similar to acute respiratory distress syndrome (ARDS) one of the hallmarks of which is prolonged and intensive mechanical ventilation [1].

While always a source of some controversy, early tracheostomy (five to seven days post-intubation) is often utilized as a means to enhance ventilator weaning, potentially decrease intensive care unit (ICU) stay, and reducing the risk of intubation associated subglottic stenosis, within critically ill patients [2]. However, the potential high risk of coronavirus transmission both while performing tracheostomy and related to tracheostomy care has caused many to modify standard practice [3]. Current recommendations by the American Academy of Otolaryngology-Head and Neck Surgery, e.g., state that tracheotomy “should not take place sooner than two to three weeks from intubation and, preferably, with negative COVID-19 testing [4].” Additionally, a 2020 systematic review of available guidelines by Heyd et al recommended delaying tracheostomy in COVID-19 positive patients until negative testing has been achieved. However, they also noted that tracheostomies may still be important in the management of COVID-19 patients with indications including prolonged ventilation and limited resources (i.e., ventilators, ICU beds, sedative restraints) [5].

Morbid obesity (BMI>34.9 + at least one obesity-related health condition or BMI>39.9) poses a unique clinical challenge, in which a more aggressive approach to tracheostomy may be warranted. In a 2013 retrospective study utilizing the Nationwide Inpatient Sample database, hospitalized morbidly obese patients were 1.37 times more likely to receive invasive mechanical ventilation and thus more likely to require tracheostomy compared to non-obese patients [6]. Additionally, there is a growing body of clinical research that has begun to show that obesity is independently associated with increased risk of intubation and mortality in patients with COVID-19 [7-13].

In the absence of COVID-19, early tracheostomy has been associated with a lower incidence of hospital-acquired pneumonia, tracheal stenosis, reduced duration of mechanical ventilation, and decreased ICU length of stay in the morbidly obese patient population [14,15]. Obesity seems to increase upper airway collapsibility both by increasing mechanical load on the upper airway and by reducing lung volume [16]. Morbidly obese patients thus seem particularly susceptible to prolonged mechanical ventilation when critically ill and seem more likely to benefit from tracheostomy placement when compared to those with a normal BMI [5,6]. This seems especially true in the setting of COVID-19, with a recent study showing that intubated patients with COVID-19 and BMI >30 had a significantly decreased chance of extubation compared to those with a BMI<30 [17]. Additionally, there is preliminary clinical evidence that suggests high rates of overweight status and obesity (BMI>30) in patients with severe COVID-19 disease requiring intubation and tracheostomy [18-20]. However, a study citing obesity as an indication for surgical tracheostomy and an obesity rate of 90% in their population of ten patients observed several benefits of tracheostomy including reduced ICU length of stay, earlier spontaneous breathing, and faster rehabilitation [18].

The primary objective of this paper was to determine whether or not early tracheostomy results in better outcomes for morbidly obese patients with COVID-19 using preliminary clinical results. The secondary objectives were to determine the safety of early tracheostomy in regards to disease transmission and discuss our institution’s current practices with reference to intensive care literature.

## Materials and methods

We performed a retrospective chart review of all patients with COVID-19 who required tracheostomy from 03/01/20 to 01/04/21. Only morbidly obese patients, defined as BMI ≥35, met inclusion criteria for our case series. We collected data including patient gender, age, BMI, pre-existing comorbidities, extracorporeal membrane oxygen (ECMO) status, length of endotracheal intubation, tracheostomy tube size, vent weaning status, number of days post-tracheostomy, subglottic stenosis, and disposition. Due to the small number of suitable study participants identified, only descriptive statistics were conducted for this article.

This study was approved by the institutional review board of the University of South Florida.

## Results

Patient demographics

Of the 31 patients with COVID-19 who received tracheostomy at our institution, we identified nine with a BMI of ≥35. Six of these patients were female with a mean BMI of 41.47 and a mean age of 54 years. Six of these patients had at least one pre-existing medical comorbidity other than morbid obesity. Five had hypertension, four had asthma two had diabetes mellitus and two had heart disease. Three patients received ECMO treatment.

Outcomes

The average number of days with endotracheal intubation prior to tracheostomy was 13.1 with four patients undergoing tracheostomy at less than two weeks. At the time of this publication, 55.6% of included patients were successfully weaned from ventilation. There was only one event of subglottic stenosis in a patient who received tracheostomy after two weeks of endotracheal intubation. Ultimately, there was a 44.4% mortality rate following tracheostomy. These outcomes are detailed in Table 1.

**Table 1:** Medical profile and clinical course of morbidly obese tracheostomy patients with COVID-19. COVID-19: coronavirus disease 2019, HTN: hypertension, DM: diabetes mellitus, HD: heart disease, ECMO: extracorporeal membrane oxygen ETT: endotracheal tube, M: male, F: female

	Gender	Age	BMI	Comorbidities	ECMO Status		No. days intubated (ETT)	Tracheostomy tube size	Weaned off ventilator	No. days from tracheostomy to ventilator wean	Subglottic stenosis	Length of stay	Disposition
	M	65	35.70	HTN, DM, HD		No	18	8	Yes	27	No	55	Rehab Facility
	M	36	35.90			Yes	17	8	No		No	52	Deceased
	F	54	37.22	Asthma, HTN		No	13	8	Yes	10	No	41	Rehab Facility
	F	83	37.93	Asthma, HTN, DM, HD		No	12	8	No		No	26	Deceased
	F	53	39.10	HTN		No	8	8	Yes	13	No	30	Home
	F	38	41.80	Asthma		Yes	16	8	No		No	49	Deceased
	F	55	44.32	HTN, Asthma		No	16	8	Yes	8	No	37	Home
	F	47	45.31			No	14	8	Yes	7	Yes	46	Rehab Facility
	M	55	55.96			Yes	4	8	No		No	23	Deceased
Mean		54.0	41.47				13.1			13.0		39.89	

Personnel follow-up

To this date, none of our personnel have developed symptoms of or tested positive for COVID-19 after taking part in these procedures.

## Discussion

Obesity is a well-documented risk factor for severe COVID-19 illness. This has certainly been identified within the COVID-19 ICU at our institution, with a mean BMI of 32.20 across all COVID-19 patients requiring tracheostomy. Our cohort of morbidly obese COVID-19 patients has been difficult to wean off mechanical ventilation, even after the most acute phase of their respiratory illness. This observation is in line with the evolving set of published clinical data [17-20]. Hence, tracheotomy is performed after seven days of mechanical ventilation and daily failed weaning trials in the morbidly obese COVID-19 patient population. However, due to the small population size of our study, it is impossible to draw any definitive conclusions about the potential benefits of early tracheostomy in this patient population. We do hope that this data will contribute to the growing pool of clinical data regarding tracheostomy timing and indication in the morbidly obese COVID-19 patient population [18-20].

A recent Italian case series described a successful tracheostomy performed in a 56-year-old severe acute respiratory syndrome coronavirus 2 (SARS-CoV-2) positive patient after five days of endotracheal intubation. None of the involved medical or nursing staff presented with symptoms 20 days after the procedure [21]. In fact, across a growing body of case series, there has been no reported evidence of disease transmission to involved healthcare workers regardless of time to tracheostomy [18,22-25]. Additionally, recent data has shown that the positive testing window, up to 37 days after symptom onset, might not correlate with infectivity which may precipitously drop eight days after developing symptoms [26]. Tracheostomy has thus been utilized toward extubation successfully at increasingly earlier periods.

Even with the recent approval of a SARS-CoV-2 vaccine and its administration to healthcare workers, there is still insufficient evidence to definitively conclude that this vaccine and those currently in development will prevent disease transmission. While protecting from systemic viral replication via immunoglobulin G (IgG)-mediated humoral immunity, injectable vaccines may not effectively prevent mucosal replication which requires local production of immunoglobulin A (IgA) antibodies. Therefore, it is still unknown if these vaccines can prevent viral transmission through asymptomatic viral shedding [27]. Despite the continued risk of viral transmission, we believe tracheostomy can be performed safely in the COVID-19 patient. At the beginning of the pandemic, our institutional tracheostomy team (composed of otolaryngologists, critical care/pulmonology physicians, respiratory therapists, nursing staff, and speech therapists) devised a working tracheostomy guideline. This is in line with previously published reports but includes careful communication among all key staff prior to procedure onset. We have chosen to perform all tracheostomies at the bedside within a negative pressure isolation unit, to prevent patient manipulation and limit the potential for viral aerosolization. Our COVID-19 unit has been engineered to limit patient contact as much as possible, and thus essential machinery to run ventilators and IV lines/drips are outside of the room. This allows tracheostomies to be performed only with an attending ICU physician, otolaryngology attending, and senior otolaryngology resident in the room. A standard procedure for donning/doffing personal protective equipment (PPE) is used and supervised by nursing staff and infection control providers. PPE includes a water-impermeable gown and hood, N95 mask, goggles or face shield, foot covering, and multiple layers of surgical gloves. This protocol is in line with current published guidelines for performing tracheostomy in patients with COVID-19 [28,29].

For morbidly obese patients with difficult anatomy, an open tracheostomy has been uniformly performed, generally with the addition of a cervical lipectomy. Complete paralysis is utilized throughout the procedure. Once the trachea is exposed a period of pre-oxygenation is performed to limit the potential of desaturation. Prior to entering the airway and during any airway manipulation, ventilation is halted and not restarted until a closed circuit is achieved. This is maintained for routine trach care by nursing staff when possible (e.g. tracheostomy suctioning). Patients are maintained in a negative pressure environment with full precautions and without tracheostomy exchange until at least two negative COVID-19 tests have been performed. After this point, they are managed using a standard institutional protocol.

## Conclusions

A growing body of literature suggests tracheostomy may promote earlier ventilator weaning in this patient population, which reduces the length of ICU stay and thus conserve valuable resources in a global pandemic. Although the population size of this study was too small to draw any conclusions, we hope the data presented here will effectively add to the growing pool of clinical data for this patient population. The risk to patients and staff is minimal when the procedure is carried out as described. Therefore, it may be possible to safely perform tracheostomy both in patients who have not yet tested negative for SARS-CoV-2 and also before a two to three-week delay following endotracheal intubation. In addition to following the current guidelines and precautions when performing tracheotomy in patients with COVID-19, we suggest that early tracheotomy be considered on a case-by-case basis.
